# Does undersizing of the tibial component in unicompartmental knee arthroplasty increase the risk of fracture? A biomechanical study

**DOI:** 10.1186/s43019-025-00299-w

**Published:** 2025-11-17

**Authors:** Julius Watrinet, Sabrina Sandriesser, Philipp Blum, Peter Augat, Marianne Hollensteiner, Rolf Schipp, Julian Fürmetz, Wolfgang Reng

**Affiliations:** 1 Department of Sports Traumatology and Arthroscopic Surgery, BG Unfallklinik Murnau, Murnau, Germany; 2 Department of Orthopaedic Sports Medicine, School of Medicine, Technical University Munich, Ismaninger Str. 22, 81567 Munich, Germany; 3 Institute for Biomechanics, BG Unfallklinik Murnau, Prof. Küntscher Str. 8, 82418 Murnau, Germany; 4 Institute for Biomechanics, Paracelsus Medical University, Strubergasse 21, 5020 Salzburg, Austria; 5 Klinikum Garmisch-Partenkirchen, Endogap, Joint Replacement Institute, Auenstraße 6, 82467 Garmisch-Partenkirchen, Germany

**Keywords:** Unicompartmental knee arthroplasty, Oxford, Tibial component sizing, Periprosthetic fractures, Biomechanical testing

## Abstract

**Background:**

Unicompartmental knee arthroplasty (UKA) is a common treatment for medial osteoarthritis, providing faster recovery and better joint kinematics than total knee arthroplasty (TKA). However, periprosthetic tibial plateau fractures (TPF) remain a significant complication. Undersized tibial components, especially size AA, increase TPF risk. This study aims to examine the biomechanical relation between tibial implant size and the risk of periprosthetic fractures.

**Methods:**

A biomechanical study was conducted using 16 customized synthetic bone models to simulate the effects of tibial component sizing in UKA. Proximal tibial models with components of size A and size AA were subjected to axial loading, and the maximum load to failure and cycles to failure were measured for each size. Additionally, plastic axial deformation was calculated at the maximum load level of the weakest construct. Strain patterns were compared with clinically observed fracture lines reported in previous studies.

**Results:**

Size AA had a significantly lower maximum load and cycles to failure compared with size A (1039 N ± 75 N and 9.336 ± 925 cycles versus 1140 N ± 83 N and 8.326 ± 759 cycles, *p* = 0.031). The strain patterns were consistent with those observed in clinical studies, showing a wedge-shaped distribution from the posteromedial to the anteromedial tibial plateau. Plastic deformation was less than 0.6 mm across all specimens, with no significant difference in axial displacement between the two groups (*p* = 0.64).

**Conclusion:**

Undersizing the tibial component reduces load-bearing capacity of the tibial plateau and thereby increases the risk of periprosthetic fractures. Precise implant sizing by correct sagittal resection is essential to minimize the risk of fracture in UKA.

**Experimental study:**

Type V

## Introduction

Unicompartmental knee arthroplasty (UKA) is a widely performed treatment for medial osteoarthritis, offering advantages such as quicker recovery and preservation of joint kinematics compared with total knee arthroplasty (TKA) [1–3]. However, the long-term survival rates of UKA are lower than those of TKA [4]. Tibial plateau fractures (TPF) are a rare but major complication, occurring at an incidence of 0.6–7.2% [4–7]. These fractures typically occur within the first 3 months after surgery and are often atraumatic in origin [8].

The risk of TPFs is influenced by several factors, including surgical technique, implant positioning, and component size [9–13]. Smaller implant sizes are associated with higher fracture rates [14]. Undersizing the tibial component, especially with a reduced medial cortex distance between the implant and keel, is thought to increase the fracture risk [15]. Biomechanical studies have demonstrated that both resection depth and extended sagittal cuts increase fracture risk [9, 16, 17]; however, the impact of tibial component undersizing has not been investigated.

This study aims to examine the biomechanical relation between tibial implant size and the risk of periprosthetic fractures. It was hypothesized that undersizing the tibial component, particularly size AA compared with size A, reduces the maximum load to failure under axial loading.

## Materials and methods

- Bone model

**Fig. 1:**
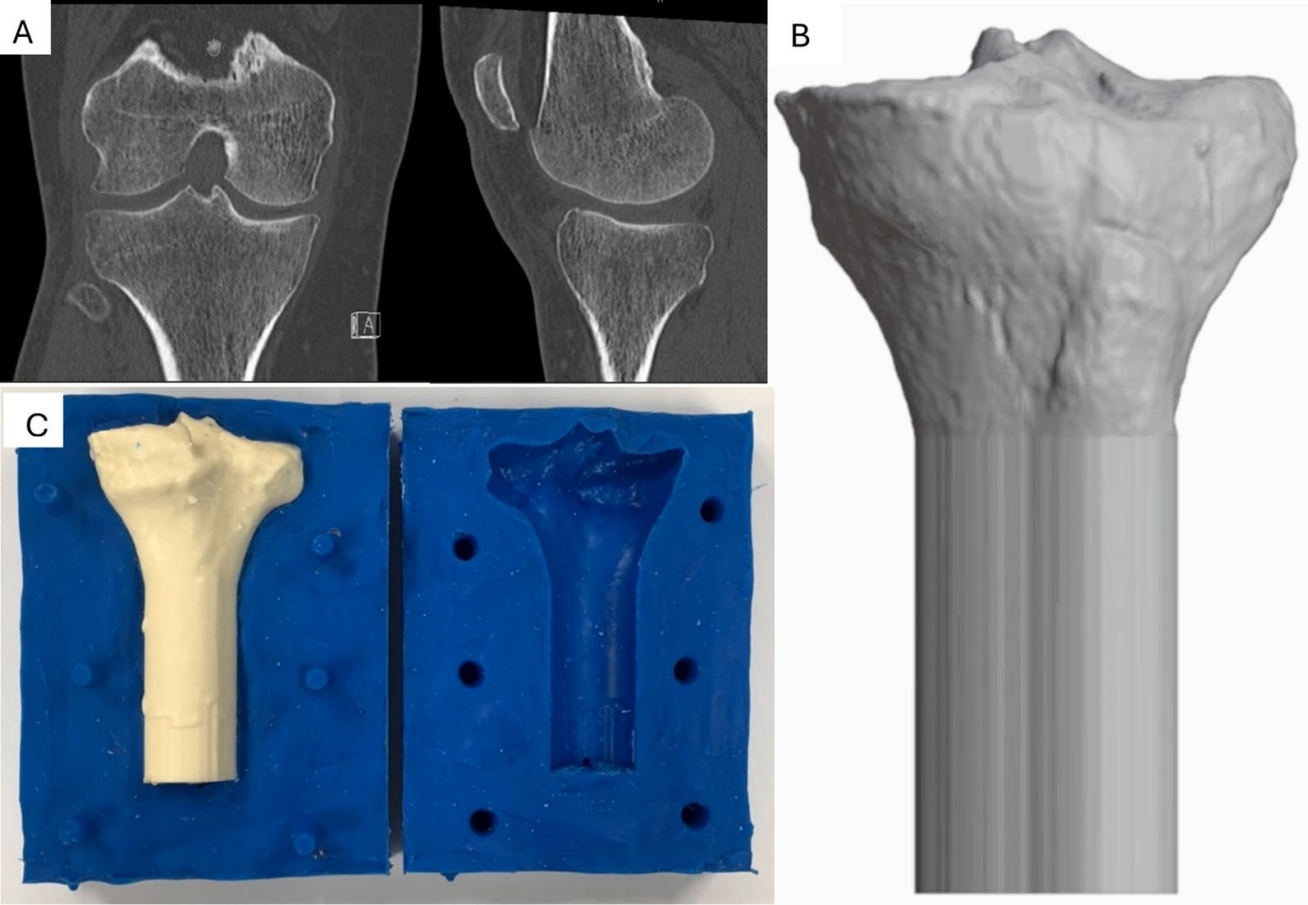
Initial CT scan of the proximal tibia in the coronal and sagittal plane (**A**). Modified 3D model (**B**). The tibial shaft was lengthened extra-anatomically for stable fixation during biomechanical testing. The 3D-printed tibial model was used to create silicon molds for the cancellous and the cortical model, respectively (**C**)

Synthetic bone models of the proximal tibia were created for this study, using a retrospectively collected anonymous computed tomography (CT) dataset. The CT dataset was chosen to fit the tibial component size A perfectly. The dataset was segmented using Dicom2Print (version 1.0.3, 3D Systems, Rock Hill, South Carolina, USA) and processed in Spaceclaim (V22 R1, Canonsburg, Pennsylvania, USA) for smoothing and scaling. A cortical model was created according to the cortical CT outline, while a cancellous bone model was sized according to the CT scan to obtain a realistic thickness of the cortical bone. The dataset was prepared for three-dimensional (3D) printing using Simplify 3D (5.1.2., Cincinnati, Ohio, USA), and a 3D model of the cortical and cancellous bone was printed using a 3D printer (N2-plus, Raise 3D, Irvine, USA) (Fig. 1).

Molds were made by embedding the 3D-printed models in silicone (MoldMax 30, Smooth-On, Inc., Easton, USA). The synthetic tibiae were cast from polyurethane treated with additives as described in detail in previous studies [18–20]. A two-step molding process enabled the fabrication of a cancellous bone encased by a cortical shell. The total length of the proximal tibia model was 12 cm.

b. Implantation

**Fig. 2:**
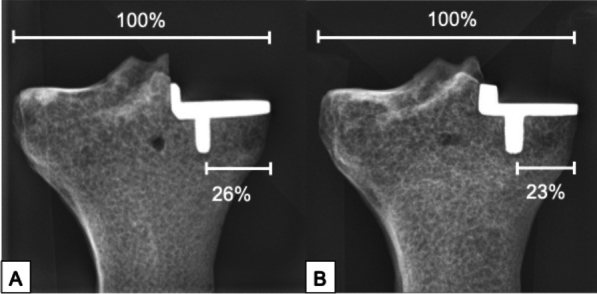
The medial knee-cortex distance was measured relatively to the tibial plateau width for tibial implant size A (**A**) as well as size AA (**B**)

The cementless implantation of the tibial component of the Oxford system (Oxford partial knee, ZimmerBiomet, Warsaw, USA) was performed by an experienced orthopedic surgeon according to the surgical technique described by the manufacturer [21]. A 3D-printed guide was used to reproducibly align the instruments in the coronal and sagittal plane for each specimen. Based on the mediolateral tibial plateau width and implant characteristics, the implantation was designed to achieve a medial keel-cortex distance to tibial plateau-width ratio of 26% for size A and 23% for size AA in the frontal view, following flush implantation at the medial cortex, in accordance with clinical findings (Fig. 2) [15, 22].

Keel preparation was accomplished by a reciprocating saw blade (GominaAG, Niederwald, Switzerland) to minimize fracture risk by surgical technique [23]. The tibial components of sizes A and AA were implanted in groups of eight each. Undersizing was defined according to Watrinet et al. as a non-matching pair of components where the tibial component is too small (Table 1) [14].

c. Biomechanical testing

**Table 1:** Matching implant sizes

Femoral component size	XS	S	M	L
Tibial component size	AA	A/B	C/D	E/F

L, large; M, medium; S, small; XS, extra-small

Eight specimens for each tibial component size were tested. Each specimen was distally embedded for 6 cm in custom made aluminum pots using a fast cast resin (010 A/B + Filler DT 082–1, Gößl + Pfaff GmbH, Karlskron/Brautlach, Germany). The tibial component was aligned horizontally in the frontal and sagittal view. The specimen was clamped to the testing machine (Instron E3000, Instron Structural Testing, High Wycombe, UK) (Fig. 2). The medial tibial plateau was loaded through a fitting tibial mobile bearing by a customized artificial medial femoral condyle of the Oxford type, which was fixed to the center of the machine actuator.

The load protocol started with three quasi-static ramps from 50 to 250 N at a speed of 10 mm/min. This was followed by cyclic sinusoidal loading with a stepwise increasing axial load. The lower load level was kept constant at 50 N. The upper load level started at 250 N and was increased for 50 N every 500 cycles. For better resolution, at loads higher 900 N the upper load level was increased by 10 N every 100 cycles. Axial load, load cycles, and actuator displacement were recorded during testing. Tests were terminated when a fracture occurred and maximum load and cycles to failure were documented.

d. Optical strain measurement

Von Mises strain on the medial surface of the synthetic bone was measured using digital image correlation with a sprayed stochastic pattern and an optical measurement system (ARAMIS, 6 M, Carl Zeiss GOM Metrology GmbH, Braunschweig, Germany). Images were captured at the unloaded and loaded condition at each load level. Images were analyzed descriptively regarding fracture patterns and visually compared with the fractured sample and clinical observations.

e. Statistical analysis

The sample size was calculated using G*Power 3.1.9.6 on the basis of data from preliminary testing [24]. A minimum sample size of five per group was required to achieve a power of 95% (effect size = 2.7, alpha = 0.05) for a given mean difference of the maximal load to failure of 130N ± 50 N based on a pretest.

All data were expressed as means and standard deviation (SD). Normal distribution was assessed using the Kolmogorov–Smirnov test. To analyze differences between two groups, non-paired Mann–Whitney *U* test was employed for numerical values. The plastic axial deformation was measured after 7000 cycles and a load of 900 N, which corresponds to the maximum load level of the weakest construct. The significance level was set at 0.05. The analysis was performed using R Studio Version 2023.03.1 (Posit Software, PBC, Boston, USA).

## Results

**Fig. 3:**
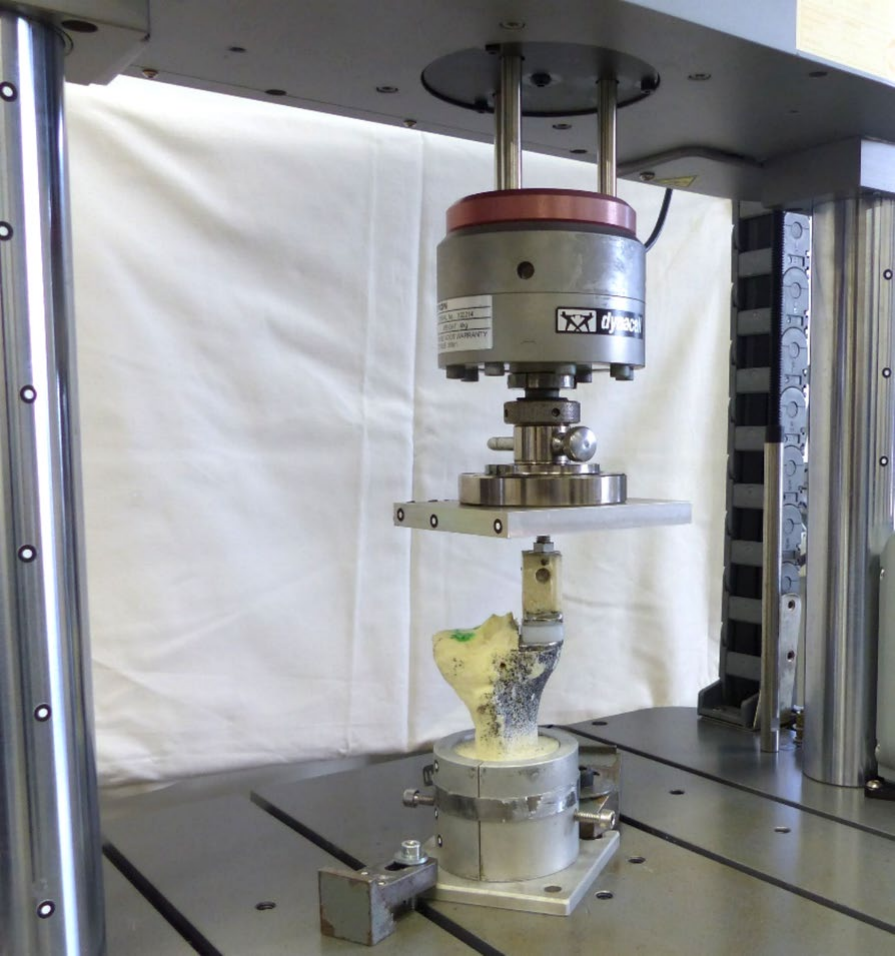
The biomechanical test setup with a synthetic tibial specimen placed in a custom-made aluminum pot and the implanted tibial component. Axial load was applied through a customized artificial medial femur condyle. On the medial side a stochastic pattern was sprayed for optical strain measurement

The analysis of the maximum load to failure revealed a significant difference between the two implant sizes (*p* = 0.031, Fig. 3). The mean maximum load to failure for the size A implant was 1140 ± 83 N (range 950–1220 N), while the size AA implant exhibited a lower mean maximum load to failure of 1039 ± 75 N (range 940–1160 N) (Table 2).

**Table 2:** Comparison of the maximum load to failure and the number of cycles to failure of two tibial implant sizes (A and AA)

Tibial component size	A (*n* = 8)			AA (*n* = 8)			*p*-Value
	Mean	SD	Range	Mean	SD	Range	
Maximum load (*N*)	1140	83	950–1220	1039	75	940–1160	0.031
Maximum cycles	9366	925	7400–10,100	8326	759	7393–9572	< 0.001

Regarding the number of cycles to failure, implant size A exhibited a significant higher mean cycle count, with an average of 9366 ± 925 cycles (range 7400–10,100 cycles), compared with size AA, which reached a mean of 8326 ± 759 cycles (range 7393–9572 cycles, *p* < 0.001).

**Fig. 4:**
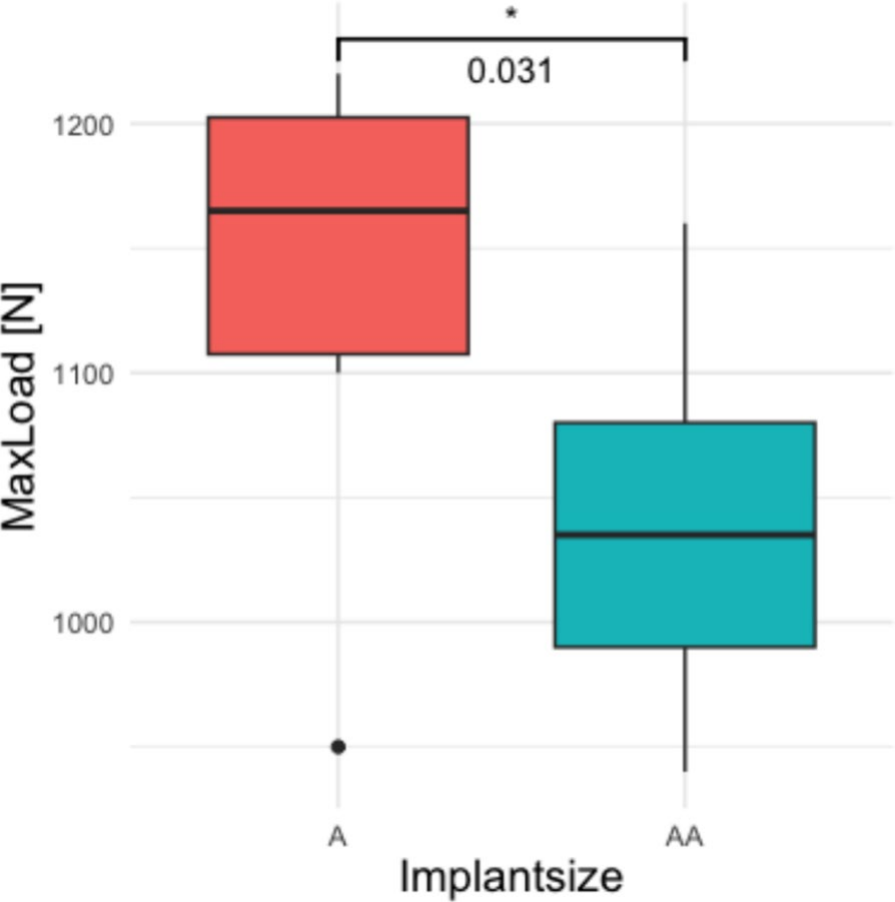
The box plot compares the maximum load to failure for implant sizes A and AA. The median for size A is higher (1165 N) than for size AA (1035 N). The interquartile ranges (IQR) are similar, but size A shows greater variability (950 N–1220 N) compared with size AA (940 N–1160 N)

**Fig. 5:**
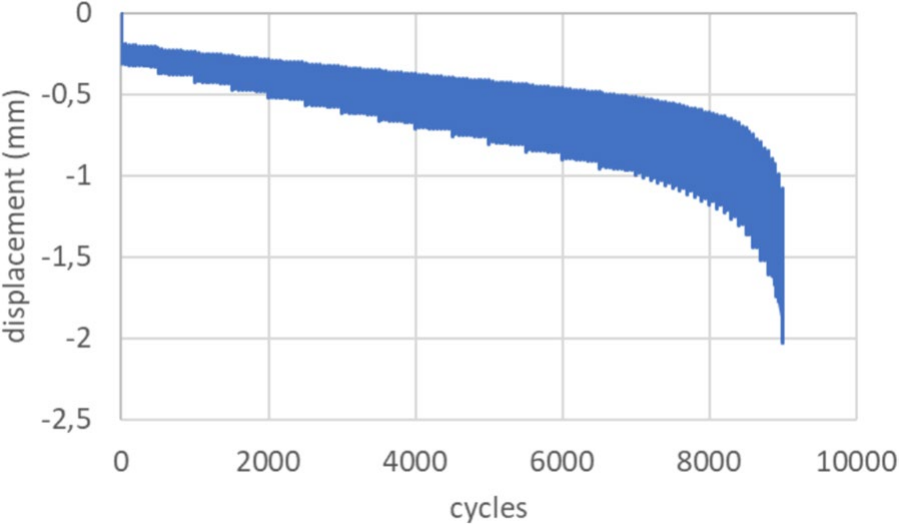
Exemplary axial displacement (mm) over load cycles. Constant subsidence of the implant until failure

Axial displacement of the implant over load cycles indicates a consistent increase in displacement as the load cycles progress, suggesting a gradual plastic deformation of the implant seat (Fig. 4). Up to 7000 cycles, which corresponds to 900 N, no significant difference in plastic deformation was observed between implant sizes A and AA (0.55 ± 0.14 mm for size A and 0.57 ± 0.11 mm for size AA, *p* = 0.64). Beyond this point, displacement increased more rapidly as the implants approached failure (Fig. 5).

**Fig. 6:**
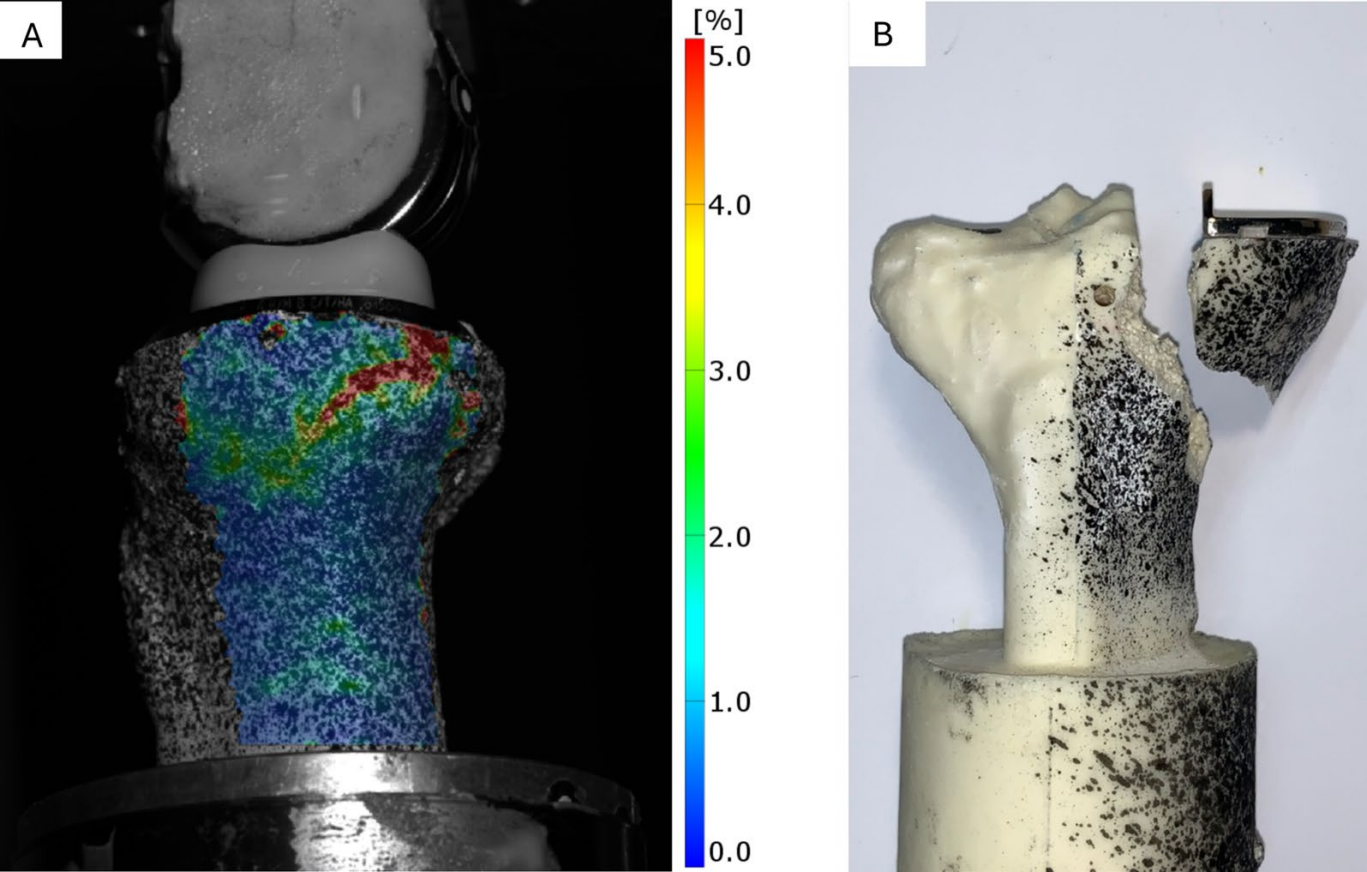
Exemplary medial view on the wedge-shaped strain pattern (**A**). Image was taken just before failure and shows the von Mises strain (blue = 0%; red = 5%). Frontal view on a fractured sample (**B**). The stochastic pattern was sprayed only on the medial side for optical measurement

The strain patterns observed in all specimens were consistent, with maximum strains localized at the sites where the fracture lines were later observed (Fig. 6). The strain distribution followed a wedge shape, extending from the posteromedial region, around the convex junction between the tibial plateau and shaft, and toward the anteromedial aspect. This optical pattern was similar to fracture types II and V as described by Burger et al. [8].

## Discussion

The primary finding of this study is that undersizing the tibial component, particularly implant size AA, results in a lower maximum load to failure compared with implant size A. The observed strain distribution followed a U-shaped pattern, extending from the posteromedial to the anteromedial aspect of the tibial plateau, which is consistent with fracture patterns seen in clinical cases of TPF [8, 15]. Additionally, plastic deformation of less than 0.6 mm occurred in all specimens after 7000 cycles, indicating that slight subsidence of the implant is common but does not significantly affect the early failure mechanisms or fracture occurrence under the tested conditions.

Our results suggest that undersizing the tibial component, specifically with size AA, increases fracture risk. The undersizing of the tibial component leads to a decreased medial keel-cortex distance. Thereby the supporting surface area of the tibial plateau is reduced, which compromises the mechanical integrity of the tibial plateau. During tibial resection, the vertical cut is made close to the anterior cruciate ligament (ACL) insertion, often leading to a sparing resection that restricts the size of the tibial component that can be used. This might lead to an undersizing of the tibial component further exacerbating the biomechanical overload, resulting in collapse of the medial tibial plateau, as seen in our study and supported by previous research [10, 25]. We assume that the increased fracture rates of tibial component size AA are associated with the increased incidence of undersizing in this component size. The implantation of correctly sized implants is associated with superior biomechanical results and therefore lower risk of TPF, leading to the assumption that tibial component size AA can safely be implanted if it matches the femoral component.

A key strength of this study is the use of a custom-made bone model, which more accurately replicates the anatomy of the tibial plateau compared with commercially available synthetic bone models. The biomechanical results observed in this study are more consistent with failure loads from cadaveric studies, ranging from 2000 to 4000 N, than the 5000 to 7000 N range seen in commercially available synthetic bone models [9, 16, 26, 27]. The individual design allowed for targeted research on individual implant sizes, a flexibility not offered by standard bone models. The applied load protocol, consisting of approximately 7000–10000 cycles, was designed to simulate the initial loading conditions following surgery [28]. Load transfer occurred via the mobile-bearing inlay, which effectively minimized shear forces and ensured the application of a purely axial load. Optical strain measurement was utilized solely to compare the fracture patterns, without analyzing or comparing the magnitude of the strain itself. This approach allowed for a focused evaluation of the fracture behavior while minimizing confounding influences from varying strain levels.

As the number of cycles to failure is reduced in tibial component size AA, fatigue damage in the pathomechanism of TPF might be considered. Bone fatigue arises from the accumulation of microcracks under repetitive submaximal loading, ultimately leading to structural collapse once a critical damage threshold is reached [29]. At the same time bone subjected to higher stress magnitudes fails after markedly fewer cycles, while lower stresses can be tolerated longer [30]. The earlier failure observed with tibial component size AA indicates a reduced tolerance to maximum load, suggesting that acute overload rather than progressive bone fatigue may represent the dominant failure mechanism.

The results of this biomechanical study emphasize the importance of careful implant sizing in UKA, suggesting that undersizing the tibial component increases the risk of periprosthetic fractures. Surgeons should prioritize using the largest tibial component that fits within the patient’s anatomy, ensuring the resection is as close as possible to the ACL insertion, which could otherwise limit implant size. Additionally, thorough preoperative planning is essential to ensure optimal tibial implant sizing, which provides adequate support and minimizes the risk of fractures after surgery.

This study has several limitations that should be considered when interpreting the results. While synthetic bones provide a controlled environment, they lack biological factors such as bone remodeling and mineralization, which may affect fracture patterns in vivo. Additionally, the study does not account for patient-specific factors such as osteoporosis or anatomical variations, which can influence fracture risk in clinical settings. The focus on a single implant design and simplified loading conditions limits the generalizability of the findings to other implant systems or real-life joint mechanics. Moreover, an undersizing of the tibial component was chosen specifically for size A, as it was the most common size associated with high fracture rates in clinical studies, which limits the conclusions that can be drawn regarding other implant sizes [15, 31]. Finally, the study’s in vitro nature does not address long-term outcomes, such as the effects of implant wear or changes in bone structure.

## Conclusion

Undersizing the tibial component, particularly to size AA, reduces load-bearing capacity of the tibial plateau and thereby increases the risk of periprosthetic fractures. Precise implant sizing by correct sagittal resection is essential to minimize the risk of fracture in UKA.

## Data Availability

Data cannot be made available.

## Abbreviations

UKA: Unicompartmental knee arthroplasty
TKA: Total knee arthroplasty
TPF: Tibial plateau fracture
CT: Computed tomography
ACL: Anterior cruciate ligament
SD: Standard deviation
IQR: Interquartile range
3D: Three-dimensional
